# Semmelweiss : His Life and His Doctrine

**Published:** 1910-06

**Authors:** 


**Semmelweiss: His Life and his Doctrine.** By Sir WILLIAM J. SINCLAIR, M.A., M.D. Pp. x, 369. Manchester: At the University Press. 1909.

This account of the life and struggles of a man who must be ranked among the greatest of the pioneers of medical discovery should be read by every member of the profession. It gives the history of a discovery which as an instance of logical deduction and research work patiently carried out under circumstances of unusual difficulty, owing to official prejudice and professional jealousy, has been seldom equalled. It may be objected that the work suffers from prolixity, and that personal and political quarrels receive undue attention. We think, however, that without these details, petty as many of them may seem, it is hardly possible to appreciate the almost insurmountable obstacles placed in the way of Semmelweiss by political influence, the personal jealousy of his superiors, and too often by the deliberate insubordination of inferiors. That Semmelweiss did not push his doctrine by the wider publication of his discovery and its results is a fact which cannot be regretted too deeply, as its acceptance would undoubtedly have saved many thousands of lives. At the same time, his diffidence is not surprising when we find that he was regarded by practically the whole of the continental obstetric world as a heretical, if not a blasphemous innovator, whose discovery, supported as it was by reasoning which to-day seems absolutely conclusive, to the official mind of his time was not deemed worth detailed examination.

Every medical practitioner who desires to read an account of a discovery made by continuous, accurate, clinical observation and logical reasoning from facts should read this volume as an illustration of the way in which research should be conducted. The gradual evolution of the doctrine and its dramatic confirmation by the tragic death of Kolletshka is a splendid illustration of continuous cumulative observation and accurate reasoning. The details given of his life-long battle against every kind of difficulty, from open opposition of equals and superiors to the veiled hostilities of ignorant and treacherous subordinates, are a lesson to all of us to-day that in the practice of new principles, even if based on actual evidence and reasoning, opposition of the kind mentioned must be expected and overcome, and also that the natural conservative instincts of humanity generally impose much strain on the loyalty of subordinates who have to carry out the details involved in the practice of new principles. Another lesson we may read in this volume is not to be too ready to condemn as new-fangled notions methods to which we have been hitherto unaccustomed, and to remember how the conclusions of Semmelweiss, though treated with contempt by his contemporaries, have since been proved to be absolutely incontrovertible. The most notable feature in the "doctrine" is this, that if Semmelweiss, instead of stating that puerperal fever was due to contagion from dead or decaying animal matter, had been able to state that it was due to the *causes* of decay and death in animal matter, namely micro-organisms, the position of his doctrine would have been entirely unassailable. As the author says, the distinction between these two points is a mere logomachy, and speaking broadly, the doctrine of Semmelweiss is as true to-day as it was at the time of its discovery.

